# Interoceptive training impacts the neural circuit of the anterior insula cortex

**DOI:** 10.1038/s41398-024-02933-9

**Published:** 2024-05-23

**Authors:** Ayako Sugawara, Ruri Katsunuma, Yuri Terasawa, Atsushi Sekiguchi

**Affiliations:** 1 Department of Behavioral Medicine, National Institute of Mental Health, National Center of Neurology and Psychiatry, Tokyo, Japan; 2 School of Psychology, The University of Queensland, St Lucia, QLD 4072 Australia; 3 Department of Psychology, Keio University, Minato-ku, Tokyo Japan

**Keywords:** Neuroscience, Human behaviour

## Abstract

Interoception is the perception of afferent information that arises from anywhere and everywhere within the body. Recently, interoceptive accuracy could be enhanced by cognitive training. Given that the anterior insula cortex (AIC) is a key node of interoception, we hypothesized that resting functional connectivity (RSFC) from AIC was involved in an effect of interoceptive training. To address this issue, we conducted a longitudinal intervention study using interoceptive training and obtained RSFC using fMRI before and after the intervention. A heartbeat perception task evaluated interoceptive accuracy. Twenty-two healthy volunteers (15 females, age 19.9 ± 2.0 years) participated. After the intervention, interoceptive accuracy was enhanced, and anxiety levels and somatic symptoms were reduced. Also, RSFC from AIC to the dorsolateral prefrontal cortex (DLPFC), superior marginal gyrus (SMG), anterior cingulate cortex (ACC), and brain stem, including nucleus tractus solitarius (NTS) were enhanced, and those from AIC to the visual cortex (VC) were decreased according to enhanced interoceptive accuracy. The neural circuit of AIC, ACC, and NTS is involved in the bottom-up process of interoception. The neural circuit of AIC, DLPFC, and SMG is involved in the top-down process of interoception, which was thought to represent the cognitive control of emotion. The findings provided a better understanding of neural underpinnings of the effect of interoceptive training on somatic symptoms and anxiety levels by enhancing both bottom-up and top-down processes of interoception, which has a potential contribution to the structure of psychotherapies based on the neural mechanism of psychosomatics.

## Introduction

Interoception is the perception of afferent information that arises from any point within the body [1, 2]. Interoception refers to the sensation of physiological states inside the body, such as breathing, heartbeat, and movement of the intestinal tract, and is considered a necessary function for maintaining homeostasis [1, 2]. Interoceptive signals are concentrated in the insular cortex of the brain, and through projection to the amygdala, it is believed to play a central role in various emotional experiences [3].

Interoceptive dysfunction has been observed in various stress-related diseases [4]. Abnormalities in interoception are known to be correlated with anxiety tendencies and are also assumed to be associated with the physical symptoms of stress-related diseases. Also, interoception was associated to neuroticism [5, 6], a personality trait known to be a vulnerability factor for mental illnesses such as depression and anxiety disorders [7]. Past studies suggested patients with panic disorder were hypersensitive to body signal [8–10], as well as irritable bowel syndrome [11]. On the other hand, eating disorders like anorexia and bulimia nervosa showed opposite types of sensitivity to the cue of hunger [12, 13]. Also, patients with somatic symptom disorders impaired their interoception [14]. Therefore, both hypo- and hyper-sensitivity of interoception seemed problematic.

The anterior insula cortex (AIC) is one of the most important nodes of interoception involved in the salience network (SN) [15, 16]. Neuroimaging findings suggest that the AIC is an important structure for encoding and representing interoceptive information [2, 3, 17–19]. Neural activation in the AIC while paying attention to their interoceptive signal was well established [20, 21]. Also, the association between neuroticism and insula activity has been reported in a previous study [22]. Given that the interoceptive nervous system consists of the AIC, the anterior cingulate cortex (ACC), and nucleus tractus solitarius (NTS) was proposed [2, 23].

Recently, interoceptive accuracy was able to be enhanced by a cognitive training task [24–26] using a biofeedback technique which was modified from the heartbeat discrimination task [27–29]. First, Garfinkel et al. developed a cognitive training task to enhance interoceptive accuracy by modifying the heartbeat discrimination task [24], which was used in the abovementioned studies [25, 26] as well as the current study. The effects of interoceptive training were demonstrated as a reduction of anxiety levels in both healthy and autistic individuals [25, 26]. This training effect in healthy participants led to somatic symptoms and reduced rational decision-making [25]. These training effects were replicable and trustworthy, but their neural underpinnings remained unclear. Given that the AIC is the key node of interoception, we hypothesized that interoceptive training modifies brain circuits with the AIC. To address this issue, we measured resting-state brain activity before and after interoceptive training to examine the effects of interoceptive training and changes in brain circuits with the AIC. We expected that brain circuits of interoception, including the AIC and the SN, would be enhanced as a training effect.

In psychosomatic clinical practice, psychotherapy directly addresses the interoception for stress-related disorders. Interoception has been attracting attention as a therapeutic target in psychotherapy. Yoga and mindfulness in psychosomatic medicine aim to control attention to and evaluation of the interoception [30]. Treatments of panic disorder, an anxiety disorder with physical symptoms, and irritable bowel syndrome, a typical psychosomatic disorder, are also conducted to transform the interoception [31, 32]. So, interoception is a promising biomarker as a target for treating somatic symptoms in stress-related disorders. Developing the method to modify interoception and unveil its mechanism is beneficial for psychosomatic medicine.

## Methods

### Participants

Twenty-two healthy volunteers (15 females, age 19.9 ± 2.0 years) participated, including graduate and undergraduate students. Fourteen of them were also included in our previous investigation about the effects of interoceptive training on decision-making [25]. All participants have no history of psychiatric disorders. When conducting regression analysis, the strategy is that a sample size 10 times larger than the number of variables being treated is sufficient [33]. In this case, since we have only one IA score as a dependent variable, the idea is that 10 or more cases should be sufficient. Given this, we suppose that the sample size is sufficient for our study.

Written informed consent was obtained from each subject. This study was conducted in accordance with the ethical standards laid down in the Declaration of Helsinki. The experiment protocol was approved by the ethical committee at the National Center of Neurology and Psychiatry (A2018-013). This protocol has been registered in the University Hospital Medical Information Network (UMIN) Clinical Trials Registry (URL: http://www.umin.ac.jp), No. UMIN000037548.

### Procedure

The interoceptive training programs were similar to our previous investigation [25], developed in-house using matlab2012a. The interoceptive training program installed on a personal computer was provided to each participant. Participants were asked to undergo at least four time training in one week because four-time training enhanced interoceptive accuracy in the past study [24, 26]. Training on each day lasted about 40 min, but the total time depended on the self-pacing intervals between trials. All participants underwent psychological and behavioral assessments before (Pre) and after (Post) one-week period.

### Interoceptive training task

The interoceptive training task was also similar to our previous investigation [25], which was developed by modifying a heartbeat discrimination task [27–29]. In this task, subjects were presented with a series of tones generated either corresponding to their heartbeat (synchronous condition) or a delay (asynchronous condition). Each trial consisted of 10 tones presented at 440 Hz with 100 ms duration, triggered by the participant’s heartbeat, which was monitored by a pulse meter attached to an index finger. Under the synchronous condition, tones were generated at the beginning of the rising edge of the pressure wave. Under the asynchronous condition, a delay of 300 ms was inserted. Following Garfinkel et al.‘s study [24, 34], we added immediate correct or incorrect feedback for participants at the end of each trial to be able to update their heartbeat perception. The task consisted of 80 trials in a one-day session.

### Psychological assessments

#### Interoceptive accuracy

Interoceptive accuracy was estimated using the heartbeat perception task [35]. Subjects were asked to count their heartbeat three times in certain periods without taking a pulse, while their actual heartbeat was recorded by a pulse meter. Using both heartbeat data, we can calculate Interoceptive accuracy (IA) scores in the following formula.

$${\rm{IA\; score}}=1/3\,\sum {\rm{\{}}1-(|{\rm{Recorded\; count}}-{\rm{Counted\; count}}|/{\rm{Recorded\; count}}){\rm{\}}}.$$

Interoceptive sensitivity is usually assessed by the IA score, but no cutoff line is provided. However, IA scores are often around 0.6 to 0.7 in studies of normal subjects [36–38].

### State-trait anxiety

Anxiety symptom was evaluated by using State-Trait Anxiety Inventory (STAI) [39, 40]. This self-reported questionnaire consists of 20 items to measure anxiety state and traits using normal and reversed four-point Linkert scales. Greater scores indicate greater anxiety. The cutoff for state anxiety is 42 points for women and 41 points for men. The cutoff for trait anxiety is specified as 45 points for women and 44 points for men.

### Social anxiety

The anxiety traits of the participants were assessed in the following questionnaires: the Social Anxiety Disorder Scale (SADS) [41]. SADS is a Japanese questionnaire that assesses social anxiety traits on four subscales: social fear, avoidance, somatic symptoms and daily life interference.

### Neuroticism

To assess the neuroticism of the subjects, all subjects were asked to complete a 60-item Japanese version (5-point scale) of the NEO-FFI [42, 43]. The neuroticism traits were previously described as follows [44]: neuroticism, the tendency to experience negative emotions and psychological distress in response to stressors.

### Somatic symptoms

A modified somatic perception questionnaire (MSPQ) [45] assessed somatic symptoms in daily life. This questionnaire consists of 22 items to evaluate how subjects feel during the past week about somatic symptoms, including heart rate increase, pulse in the neck, butterflies in the stomach, pain or ache in the stomach, difficulty in swallowing, mouth becoming dry, and so on. Subjects were asked to check four-point Linkert scales. Greater scores indicate greater sensitivity to somatic perception.

### MRI data acquisition

MRI images were acquired using a 3-T MR scanner with a 32-channel phased array head coil (MAGNETOM Verio Dot, Siemens Medical Systems, Erlangen, Germany). Resting-state functional MRI (rsfMRI) and structural MRI data were obtained from each participant using the following MRI acquisition protocol. rsfMRI data were acquired with gradient-echo echo planar imaging (GE-EPI) for 10 min, during which participants were asked to clear their minds and to focus on a central fixation cross. We administered the Stanford sleepiness scale [46] to guarantee the wakefulness of participants during scanning. Acquisition parameters of GE-EPI were repetition time (TR) = 2500 ms, echo time (TE) = 30 ms, flip angle (FA) = 80°, voxel size = 3.3 × 3.3 × 3.2 mm^3^ (with a 0.8-mm gap), 40 axial slices, and 240 volumes. Structural MRI was acquired using a three-dimensional T1-weighted magnetization-prepared rapid gradient-echo (MPRAGE) sequence: TR = 1900 ms, TE = 2.52 ms, inversion time (TI) = 900 ms, FA = 90°, 192 sagittal slices, and voxel size = 0.98 × 0.98 × 1 mm^3^.

### Data analyses

Data from one participant who did not perform intervention tasks because of a personal reason was omitted. Behavioral data from another participant were not available because of an unexpected technical error in the tablet PC. Post-intervention MRI data from 2 participants were not obtained because of an unexpected MRI machine failure. Also, scores of interoceptive accuracy, an independent value for the MRI analyses in the present study, from one participant were omitted because of an outlier that was more or less than two standard deviations. Thus, the analysis included longitudinal MRI and psychological data from 17 participants.

### Psychological data analyses

Psychological and behavioral data were analyzed using statistical software, IBM SPSS Statistics v.25. To detect significant longitudinal changes from before and after the training, paired t-tests were conducted on indices of interoceptive accuracy and scores of anxiety symptoms and somatic symptoms. Statistical thresholds were set at *p* = 0.05 one-tailed, based on our hypotheses that indices of interoceptive accuracy and anxiety and somatic symptoms would decrease after training. Statistical thresholds were set at *p* = 0.05 one-tailed, based on our hypotheses that according to the improvement of interoceptive accuracy.

### MRI data analyses

T1w anatomical data were preprocessed using N4 inhomogeneity correction [47]. rsfMRI data were analyzed using CONN [48] with SPM12. Preprocessing includes slice-timing correction, realignment, coregistration, segmentation, normalization, and smoothing with an 8 mm full width at half maximum (FWHM) isotropic Gaussian kernel, denoising (using white matter, CSF, and realignment parameters), and motion scrubbing. Then, the time-series data were band-pass filtered (0.01–0.1 Hz).

Seed to Voxel maps was created as resting state functional connectivity (RSFC) from rsfMRT data. Right (47,14,9) and left (-44,13,1) anterior insula were set as seed ROIs. These coordinates of bilateral insula were defined by default in the CONN software. Delta images of the seed-to-voxel maps (Post–Pre) were calculated for each subject. Multiple regression analyses for the delta images using change ratio of IA scores. The statistical threshold was set at *p* < 0.001 and corrected to *p* < 0.05 for multiple comparisons using cluster size.

## Results

Table 1 shows psychological changes from before to after the interoceptive training. As for anxiety levels, it stipulates that if the score for state anxiety is below 30 for women and 31 for men, or if the score for trait anxiety is below 33 for women and 32 for men, the score is considered “low” anxiety. Therefore, from the average STAI scores, it can be read that characteristic state anxiety was a low anxiety group at baseline. Significant changes in scores of interoceptive accuracy, which is a direct training effect. Negative traits such as trait anxiety scores, social anxiety scores, and neuroticism are improved after the training. Somatic symptoms are also improved after training, similar to our previous findings.

**Table 1:** Psychological measures before (Pre) and after (Post) training.

Psychological measures	Pre (*n* = 17)	Post (*n* = 17)	
Interoceptive accuracy (IA)	0.63 ± 0.17	0.79 ± 0.14	*p* < 0.01**
Somatic symptoms (MSPQ)	5.8 ± 4.2	4.4 ± 3.3	*p* < 0.05*
Trait anxiety (STAI)	28.6 ± 11.1	27.3 ± 10.2	*p* < 0.05*
State anxiety (STAI)	20.1 ± 8.9	19.1 ± 10.1	*p* = 0.13
Social anxiety (SAD)	37.8 ± 22.9	33.7 ± 23.0	*p* < 0.05*
Neuroticism (NEO-FFI)	28.8 ± 10.7	26.4 ± 11.0	*p* < 0.005***

* *p* < 0.05, ** *p* < 0.01, *** *p* < 0.005, one-tailed paired t-test.

*IA* interoceptive accuracy, *MSPQ* modified somatic perception questionnaire, *STAI* state-trait anxiety inventory, *SAD* social anxiety disorder, *NEO-FFI* NEO-five factory inventory.

**Fig. 1:**
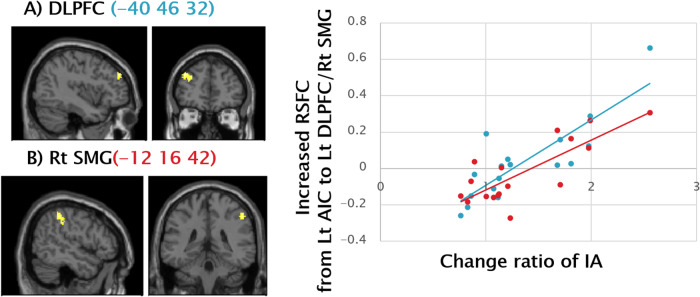
Positive Correlation between Change Ratio of IA and RSFC from Left AIC to Left DLPFC and Right SMG. Significant positive correlations were observed between the change ratio of IA and increased RSFC from the left AIC to (**A**) the left DLPFC (-40 46 32) (Pearson’s r = 0.84, *p* < 0.001), and (**B**) the right SMG (-12 16 42) (Pearson’s r = 0.79, *p* < 0.001), illustrated by the blue and the red scatter plots on the right side, respectively. Vertical axes represent increased RSFC at peak voxel in each cluster, and horizontal axes indicate a change ratio of IA (i.e., Post IA/Pre IA). The statistical threshold was set at *p* < 0.001 and corrected to *p* < 0.05 for multiple comparisons using cluster size. IA interoceptive accuracy, RSFC resting state functional connectivity, AIC anterior insula cortex, DLPFC dorsolateral prefrontal cortex, SMG supramarginal gyrus, CCN cognitive control network.

Significant positive correlations were observed between the change ratio of IA and increased resting state functional connectivity from the left AIC to left DLPFC (Fig. 1A) and the Right SMG (Fig. 1B), suggesting resting functional connectivity from the AIC to the cognitive control network (CCN) was enhanced according to the training effects.

**Fig. 2:**
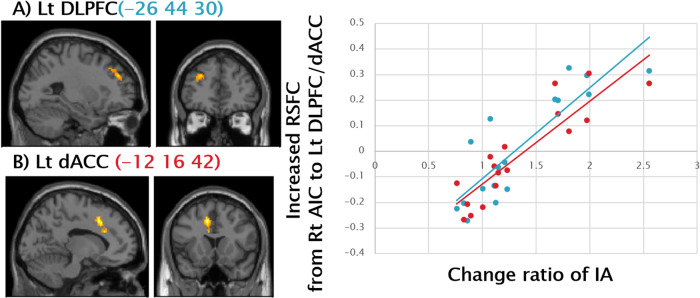
Positive Correlation between Change Ratio of IA and RSFC from Right AIC to Left DLPFC and Left Dorsal ACC. Significant positive correlations were observed between change ratio of IA (i.e., Post IA/Pre IA) and increased RSFC from the right AIC to (**A**) the left DLPFC (-26 44 30) (Pearson’s r = 0.86, *p* < 0.001), and (**B**) the left dorsal ACC (-12 16 42) (Pearson’s r = 0.89, *p* < 0.001), illustrated by the blue and the red scatter plots on the right side, respectively. The statistical threshold was set at *p* < 0.001 and corrected to *p* < 0.05 for multiple comparisons using cluster size. IA interoceptive accuracy, RSFC resting state functional connectivity, AIC anterior insula cortex, DLPFC dorsolateral prefrontal cortex, SMG supramarginal gyrus, CCN cognitive control network, SN; salience network.

Significant positive correlations were observed between the change ratio of IA and increased resting state functional connectivity from the right AIC to left DLPFC (Fig. 2A) and the left dorsal ACC (Fig. 2B), suggesting resting functional connectivity from the AIC to the CCN, as well as the SN, were enhanced according to the training effects.

**Fig. 3:**
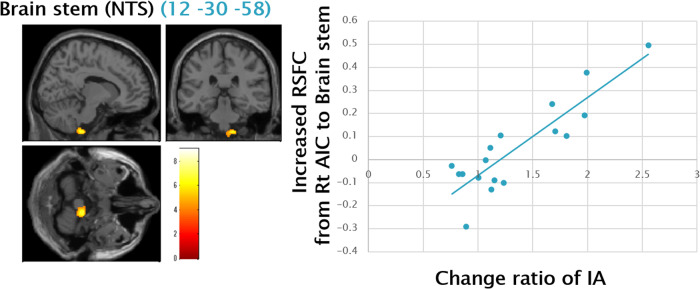
Positive Correlation between Change Ratio of IA and RSFC from Right AIC to Brain Stem. A significant positive correlation was observed between the change ratio of IA (i.e., Post IA/Pre IA) and increased RSFC from right AIC to the brain stem (12 -30 -58) (Pearson’s r = 0.87, *p* < 0.001), illustrated by the blue scatter plots on the right side. The cluster includes NTS, a key node of the vagal nerve. The statistical threshold was set at *p* < 0.001 and corrected to *p* < 0.05 for multiple comparisons using cluster size. IA interoceptive accuracy, RSFC resting state functional connectivity, AIC anterior insula cortex, NTS Nucleus tractus solitarius.

A significant positive correlation was observed between the change ratio of IA and increased resting state functional connectivity from the right AIC to the brain stem, including NTS (Fig. 3), a key node of the vagal nerve.

**Fig. 4:**
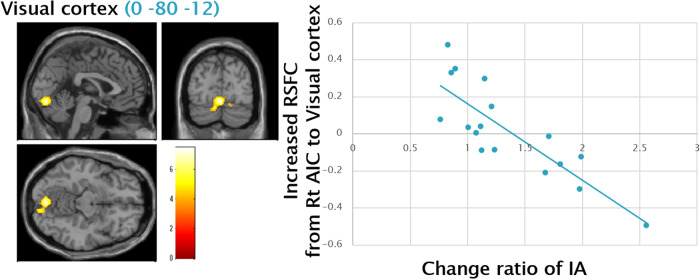
Negative Correlation between Change Ratio of IA and RSFC from Right AIC to Visual Cortex. A significant negative correlation was observed between the change ratio of IA (i.e., Post IA/Pre IA) and increased RSFC from the right AIC to a cluster including the VC (0 -80 -12) (Pearson’s r = 0.87, p < 0.001), illustrated by the blue scatter plots on the right side. The statistical threshold was set at *p* < 0.001 and corrected to *p* < 0.05 for multiple comparisons using cluster size. IA interoceptive accuracy, RSFC resting state functional connectivity, AIC anterior insula cortex, VC visual cortex.

A significant negative correlation was observed between the change ratio of IA and increased resting state functional connectivity from the right AIC to a cluster including the visual cortex (VC) (Fig. 4).

## Discussion

After the one-week interoceptive training, 1) interoceptive accuracy was enhanced, and 2) somatic symptoms, anxiety levels, and neuroticism were reduced. In line with the effect of interoceptive training, 3) RSFC between AIC and dACC/NTS and DLPFC/SMG were enhanced; instead, 4) RSFC between AIC and VC was reduced. The results suggest that neural circuits from AIC to the SN and the cognitive control network (CCN), which are corresponding to bottom-up and top-down processes of interoception, respectively, were involved in the effect of interoceptive training.

Enhanced interoceptive accuracy after training represented a direct training effect, which was a replication of our previous findings [25]. Also, how the interoceptive training reduced the somatic symptoms and anxiety levels could be explained by a concept of multiple elements of interoception [49]. These effects were also a replication of the previous findings [24–26]. Our previous article [25] discussed the exact reasons for the abovementioned interpretations. Also, a significant reduction in neuroticism was found after training, although the effect size was not so large. Given that the neuroticism has been shown to associated with interoception and the insula activity in previous studies [5, 6, 22], it is suggested that the effect of interoceptive training also influences personality traits. This notion supports the idea that interoceptive sensitivity was helpful as a biomarker for stress-related disorders [50].

The increased “bottom-up” pathway of interoception was represented by an effect of interoceptive training on elevated interoceptive accuracy, involved in the neural bases of effects of psychotherapies like yoga and mindfulness. The enhanced functional connectivity from the AIC to the SN and node of the vagal nerve represented the enhanced bottom-up process of interoception due to the training. The AIC and the dorsal ACC are vital nodes of the SN [2, 15, 23], and NTS is a crucial node of the vagal nerve [51, 52], which is a gateway of interoceptive inputs from the body. All of them are the main “bottom-up” pathway of interoception [30], enhanced by psychotherapies related to interoceptive training like yoga and mindfulness. After the mindfulness-based stress reduction and mindfulness training, the RSFC from AIC to the SN [53] and its activations [54] were strengthened rather than control groups. Therefore, interoceptive training is supposed to benefit subjects with hypo-sensitivity of interoception by enhancing the “bottom-up” pathway of interoception.

The enhanced “top-down” pathway of interoception was represented by an effect of interoceptive training on reduced anxiety levels and somatic symptoms, which also potentially contributed to understanding the mechanism of psychotherapies like mindfulness-based cognitive therapies (MBCT). DLPFC/SMG were principal parts of the CCN [55], which was also labeled as lateral frontoparietal network [56] or central executive network [57]. Enhanced activities in the CCN were neural representations of the therapeutic effects of psychotherapies [58]. Namely, the neural circuit of AIC and DLPFC is thought to represent the central control process of emotional regulation [59] by monitoring and manipulating emotional representations in the working memory [60]. On the other hand, the neural circuit of AIC and the parietal cortex contributes to emotional regulation by using attention control [61]. These cognitive processes include a “top-down” pathway of attention to interoception [19, 61, 62]. This pathway was also enhanced by psychotherapies related to interoceptive training. The brain circuit, including AIC and the CCN, was enhanced after the MBCT [63]. Accordingly, interoceptive training is expected to benefit subjects with hyper-sensitivity to interoception by enhancing the “top-down” pathway of interoception, as well.

The interoceptive training let participants attentional resources from exteroception to interoception. The visual network is involved in paying attention to the outside of the body, corresponding to exteroception. A significant negative correlation was observed between the change ratio of IA and increased RSFC from the right AIC to the VC. A previous study demonstrated that interoceptive attention is associated with reduced coupling between AIC and VC [19], suggesting interoceptive training trains interoceptive perceptions while at the same time changing the brain circuitry for exteroceptive perceptions. The findings corresponded to the notion that interoceptive and exteroceptive perceptions are shared in an attentional resource [23]. Given that an imbalance in interoceptive-exteroceptive processing, hyper-sensitivity of interoception, and hypo-sensitivity of exteroception induced many of the symptoms of depression [64, 65], the interoceptive training is also supposed to be effective in depressive individuals by enhancing the “top-down” pathway of interoception.

There are a few limitations to this study. First, our interpretation of training effects was inconclusive because of the study’s single-arm design. To establish a training effect, appropriate control groups are needed. Second, although the heartbeat perception task, as well as the heartbeat discrimination task, which was modified to the interoceptive training task, have been well established for assessment of interoceptive accuracy labeled as the “bottom-up” pathway of interoception [66, 67], some of the top-down factors, such as knowledge or expectations of one’s pulse rate, confounds on the task performance. Consequently, our interoceptive training task enhanced not only the “bottom-up” but also the “top-down” pathway. Third, because our participants were healthy volunteers, further studies are needed involving stress-related disorder patients with hypo- and/or hypersensitivity to interoception to evaluate the beneficial effect of the interoceptive training.

## Conclusions

Our results suggested that the interoceptive training enhanced the interoceptive accuracy and improved anxiety and somatic symptoms, represented by the enhanced neural circuits of the AIC to the SN and the CCN, respectively. The findings provided a better understanding of neural underpinnings of the effect of interoceptive training on somatic symptoms and anxiety levels by enhancing both bottom-up and top-down processes of interoception, which has a potential contribution to the structure of psychotherapies based on the neural mechanism of psychosomatics. Interoception is a promising biomarker of somatic symptoms in stress-related disorders. Developing the method to modify interoception and unveil its mechanism is beneficial for psychosomatic medicine.

## Data Availability

Data from participants who agreed to the public distribution of data are available from the corresponding author upon reasonable request.
